# Controlled Clinical Studies of Combined Oral Contraceptives for Dysmenorrhea in China: A Systematic Literature Review

**DOI:** 10.1177/26884844251379378

**Published:** 2025-09-22

**Authors:** Ye Zhang, Sisi Chen, Chenxuan Wei, Menglin Qi, Sunying Zhang, Yeping Yang, Hong Xu

**Affiliations:** ^1^ Department of Gynecology, International Peace Maternity & Child Health Hospital, Shanghai Jiao Tong University School of Medicine, Shanghai, China.; ^2^ Medical Affairs and Outcomes Research, Organon Research and Development, Organon (Shanghai) Pharmaceutical Technology Co., Ltd., Shanghai, China.

**Keywords:** combined oral contraceptive, dysmenorrhea, methodological review, pain relief, adverse event

## Abstract

**Background:**

Combined oral contraceptives (COCs) can relieve dysmenorrhea, but utilization is low in Chinese women. This systematic literature review was conducted to summarize the design of clinical studies and the effectiveness and safety of COCs for dysmenorrhea in Chinese women.

**Methods:**

The PubMed, EMBASE, CNKI, Wanfang, VIP, Chinese clinical trial register, and ClinicalTrials.gov databases were searched for randomized controlled trials (RCTs), non-RCTs, and cohort studies investigating COCs for dysmenorrhea in the Chinese population. A narrative synthesis and descriptive statistics were used to summarize the clinical study designs, intensity of dysmenorrhea symptoms, and safety of COCs for dysmenorrhea in Chinese women.

**Results:**

Twenty-eight clinical studies (24 RCTs, 2 non-RCTs, 2 cohort studies) with 3409 patients were included in this review. Primary (PD) and secondary dysmenorrhea (SD) were investigated in 9 and 18 studies, respectively, and 1 study did not specify the type. Most studies gave cyclic versus continuous COCs (92.9% [*n* = 26/28] vs. 14.3% [*n* = 4/28 studies]). Traditional Chinese medicines were common comparators (PD: 66.7% [*n* = 6/9 studies]; SD: 61.1% [*n* = 11/18 studies]). Most studies reported intensity of dysmenorrhea symptoms (*n* = 22/28), usually with the visual analogue scale pain score (59.1% [*n* = 13/22 studies]). COCs significantly reduced symptoms of dysmenorrhea in PD and SD. Abnormal menstrual bleeding was the most common adverse event (2.4%–51.4%).

**Conclusions:**

COCs are effective for PD and SD in China with an acceptable safety profile. Additional head-to-head comparative trials are needed to clarify the role of COCs versus other treatments in Chinese patients.

## Introduction

Dysmenorrhea is a cramping pain experienced by women before or during the onset of menses. Primary dysmenorrhea (PD) occurs in the absence of obvious underlying gynecologic disorders.^1^ Secondary dysmenorrhea (SD) is caused by pelvic pathology such as endometriosis.^2^ Symptoms of dysmenorrhea are mainly related to increased production of endometrial prostaglandins (PGs).^2,3^ In the last decade in China, 45.5%–86.6% of women experienced dysmenorrhea^4–8^; of these, 90% had PD.^9^ Among women with PD and SD, 56.4% and 43.6%, respectively, consulted a health care provider.^10^

The management of dysmenorrhea requires lifestyle modifications and medications, including nonsteroidal anti-inflammatory drugs (NSAIDs), traditional Chinese medicines (TCM), and hormone therapies. For PD, hormone therapies include combined oral contraceptives (COCs) and progestogen-only contraceptives. Treatment of SD is determined by the underlying pelvic pathology. Hormone therapies include gonadotropin-releasing hormone agonists (GnRH agonists) and the levonorgestrel-releasing intrauterine system (LNG-IUS).^11,12^

COCs can relieve dysmenorrhea by suppressing ovulation, inhibiting the transition from proliferative to secretory endometrium, and reducing PG levels during menses.^13^ Chinese clinical guidelines^11,14,15^ and expert consensus^16^ recommend the use of cyclic or continuous COCs for treating pain associated with menstruation and gynecologic disorders. However, the utilization of COCs for dysmenorrhea is low in Chinese clinical practice, with only 4.7% of women with dysmenorrhea using progesterone-containing drugs such as COCs.^17^

The low usage of COCs for dysmenorrhea in China is due to a lack of indication, uncertainty about effectiveness, and concerns about safety. Chinese investigators have evaluated COCs for dysmenorrhea using various study designs. One randomized controlled trial (RCT) investigated COCs versus placebo for PD in 90 adolescent patients,^18^ while a non-RCT investigated COCs versus no treatment for preventing the progression of endometriosis-related pain in 316 patients.^19^

There remains an unmet clinical need to synthesize the evidence from relevant studies to provide robust and broad conclusions about the effectiveness and safety of COCs for dysmenorrhea in the Chinese population. This systematic review aimed to fill that research gap by summarizing the designs of clinical studies and evaluating the effectiveness and safety of COCs for dysmenorrhea in Chinese women. Findings will inform Chinese clinical practice and identify opportunities for further investigation.

## Materials and Methods

The protocol for this study was registered on PROSPERO in April 2024 (registration number: CRD42024523871, https://www.crd.york.ac.uk/PROSPERO/#recordDetails). This study is reported according to the Preferred Reporting Items for Systematic Reviews and Meta-Analyses (PRISMA) 2020 statement.^20^

### Search strategy

The PubMed, EMBASE, CNKI, Wanfang, VIP, Chinese clinical trial register, and ClinicalTrials.gov databases were searched for studies investigating the effectiveness and safety of COCs for dysmenorrhea in the Chinese population. Searches were restricted to articles and protocols published or registered in English or Chinese between January 2013 and January 2024. The search strategy for each database is presented in the Supplementary Appendices SA^1^–SA^7^.

### Eligibility criteria

The Population, Intervention, Comparison, Outcomes, and Study framework was used to determine the eligibility of studies for inclusion in this review (Table 1). Key eligibility criteria were: (1) Population: Chinese women with PD or SD; (2) Intervention: COCs; (3) Comparison: other treatments, including placebo and NSAIDs; (4) Outcomes: study design, including dose and duration of COCs, comparator, PD or SD, severity of dysmenorrhea, endpoints, and length of follow-up; effectiveness of COCs assessed by symptom measures such as the visual analogue scale (VAS) pain score; and safety of COCs assessed by the incidence of adverse events (AEs); and (5) Study: RCTs, non-RCTs, or cohort studies. Eligible studies must have included ≥30 patients treated with COCs, but there were no restrictions on study setting, minimum duration of treatment, and minimum length of follow-up. Studies reported in Chinese must have been published in core journals identified by the Guide to Chinese Core Journals, the Chinese Science Citation Database, and the Key Magazine of China Technology. If multiple published reports from the same study were identified, only the publication reporting the most complete original data was included.

**Table 1.** Eligibility Criteria

Population	Chinese adolescent and adult females with primary or secondary dysmenorrhea
Intervention/exposure	Combined oral contraceptive with no limit on dosage, formulation, and duration
Comparison	Placebo, no treatment, nonsteroidal anti-inflammatory drugs, traditional Chinese medicines, acupuncture and moxibustion, combined oral contraceptive, progestogen-only contraceptive, levonorgestrel-releasing intrauterine system, GnRH agonist, combination therapy of ≥2 of the above therapies
Outcome	Study designs: Dosing scheme and duration of COC; comparator; primary or secondary dysmenorrhea; the proportion of mild, moderate, and severe dysmenorrhea; endpoints; length of posttreatment follow-up Effectiveness: Dysmenorrhea symptom scores
Study design	Randomized controlled trial, non-randomized controlled trial, and prospective or retrospective cohort study
Publication details	Type: Study protocol, conference abstracts, articles Time: Studies registered or published between January 2013 and January 2024. Language: English or Chinese Studies reported in Chinese should have been published in journals identified by the Guide to Chinese Core Journals, the Chinese Science Citation Database, and the Key Magazine of Chinese Technology

COC, combined oral contraceptive; GnRH, gonadotropin-releasing hormone.

### Study selection and data extraction

Search results were de-duplicated using Endnote 20. Then, two researchers (S.C. and M.Q.) independently screened titles, abstracts, and full texts to select eligible studies. Next, the researchers independently extracted data from the eligible studies using a data extraction template in Microsoft Excel. Data included: (1) first author; (2) year of publication/registration; (3) period of patient enrollment; (4) study design; (5) sample size of each treatment group; (6) population (type of dysmenorrhea and proportion of mild, moderate, and severe dysmenorrhea); (7) comparator; (8) dosing scheme (cyclic or continuous) and duration of COCs; (9) endpoints; (10) symptom measures; (11) mean and standard deviation of VAS pain score pre- and posttreatment; (12) AEs/adverse reactions; and (13) discontinuation and reasons. No automation tools were used for study selection or data extraction, and no study author was contacted to provide clarification on missing or unclear information. Disagreements between researchers regarding study selection or data extraction were resolved through adjudication by a third researcher (Y.Z.).

### Risk of bias assessment

Two researchers (S.C. and M.Q.) independently assessed the methodological quality of the included studies using the Cochrane Risk of Bias tool for RCTs,^21^ the Risk Of Bias In Non-randomized Studies of Interventions tool for non-RCTs,^22^ and the Newcastle–Ottawa scale (NOS) for cohort studies.^23^ Disagreements were discussed and resolved through consensus, or through adjudication by a third researcher (Y.Z.), if necessary.

### Data analysis

Results were summarized using JMP 18.0 (SAS Institute, Cary, NC). Descriptive statistics were presented as medians (ranges) for continuous variables and counts (percentages) for categorical variables.

## Results

### Study selection

**FIG. 1.**
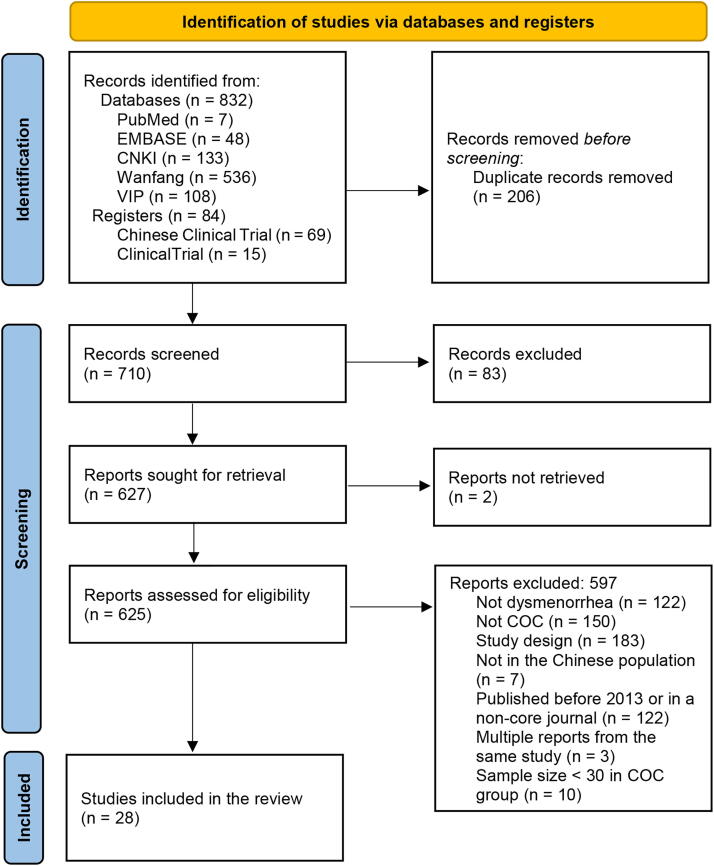
PRISMA flow diagram of study selection. We excluded ineligible records identified from registers at this stage. For records identified from databases, all reports were retrieved for full-text reviewing directly instead of abstract screening; many abstracts did not provide detailed information on methodology. PRISMA, Preferred Reporting Items for Systematic Reviews and Meta-Analyses.

The initial search identified 916 relevant reports (*n* = 832 from the databases; *n* = 84 from the registers). Titles and abstracts were screened, and 289 reports were excluded, including 206 duplicates and 83 studies that did not meet the eligibility criteria. As 2 reports were not retrievable, the full text of the remaining 625 reports was reviewed. A total of 597 reports were excluded as the studies did not enroll patients with dysmenorrhea (*n* = 122), did not investigate COCs (*n* = 150), were not controlled (*n* = 183), did not include the Chinese population (*n* = 7), were published before 2013 or in noncore journals (*n* = 122), were multiple reports from the same study (*n* = 3), or <30 patients were treated with COCs (*n* = 10). Finally, 28 studies were included in this systematic review. The PRISMA flowchart for study selection is shown in Figure 1.

### Study characteristics

The 28 studies^18,19,24–49^ included 3409 patients. Among the included studies, 24 were RCTs,^18,24–40,42–47^ 2 were non-RCTs that assigned subjects based on the order of inpatient admission^49^ and patient choice,^19^ and 2 were retrospective cohort studies.^41,48^ The sample sizes of the included studies ranged from 60 to 335. Studies investigated PD (32.1% [*n* = 9/28 studies])^18,25–32^ or SD (64.3% [*n* = 18/28 studies]),^19,33–49^ but no single study included both. One study did not specify the type of dysmenorrhea.^24^ The details of the included studies are summarized in Table 2.

**Table 2.** Characteristics of Included Studies

First author, publication year	Study design	Study period	Number of sites	Total sample size	Type of dysmenorrhea	Dose scheme and duration of COC	Comparison	Outcomes measures	Length of posttreatment follow-up
Xia, 2023^33^	RCT	2019.7–2021.6	1	65	SD	Drospirenone ethinylestradiol tablets cyclic regimen (21 + 7) for 3 months	TCM	Dysmenorrhea symptom scores; serum CA125, PGF-2α levels (before and after treatment); TCM symptom score (3 months after treatment)	3 months
Zhu, 2023^34^	RCT	2020.10–2021.10	1	62	SD	Drospirenone ethinylestradiol tablets cyclic regimen (21 + 7) for 3 months	TCM	Volume of ectopic lesions, VAS, CMSS, clinical efficacy (before and after treatment)	No follow-up
Huang, 2022^35^	RCT	2020.1–2020.10	1	64	SD	Drospirenone ethinylestradiol tablets cyclic regimen (21 + 7) for 3 months	TCM	CMSS, the adenomyosis blood stasis syndrome scale of TCM, uterine volume, serum CA125 levels, SOD, CAT, GSH-Px contents	No follow-up
Bu, 2019^25^	RCT	2017.2–2019.2	1	128	PD	Desogestrel and ethinylestradiol tablets cyclic regimen (21 + 7) for 3 months	TCM + COC	Clinical efficacy, VAS, dysmenorrhea duration, CMSS, hemodynamic indicators of bilateral uterine artery, PGF2α level, IL-10 level (before and after treatment)	3 months
Zhang, 2018^26^	RCT	2016.2–2017.9	1	102	PD	Desogestrel and ethinylestradiol tablets cyclic regimen (21 + 7) for 3 months	TCM	Clinical efficacy, dysmenorrhea score, hemorheological indexes (plasma viscosity and hematocrit) (before and after treatment)	3 months
Gong, 2018^36^	RCT	2016.5–2017.5	1	60	SD	Desogestrel and ethinylestradiol tablets for 1 month	TCM	Clinical efficacy, dysmenorrhea TCM score, serum levels of CA125, IL-6, PGF-2α (before and after treatment)	No follow-up
Ma, 2018^27^	RCT	2015.3–2017.6	1	335	PD	Desogestrel and ethinylestradiol tablets cyclic regimen (21 + 7) for 3 months	COC + TCM	Degrees of dysmenorrhea (after treatment)	6 months
Wang, 2018^37^	RCT	2014.2–2016.6	1	110	SD	Drospirenone ethinylestradiol tablets cyclic regimen (21 + 7) for 6 months	ING-IUS	VAS, menstrual blood volume, uterine volume and CA125 (before and 6 months after treatment)	No follow-up
Wu, 2016^38^	RCT	2012.1–2016.1	1	84	SD	Desogestrel and ethinylestradiol tablets cyclic regimen (21 + 7) for 3 months	TCM	Level of dysmenorrhea TCM scores and VAS, effectiveness, uterine volume and serum CA125 were observed (before and 1, 2, 3 months after treatment)	3 months
Chen, 2015^28^	RCT	2012.9–2013.9	1	98	PD	Desogestrel and ethinylestradiol tablets cyclic regimen (21 + 7) for 3 months	Placebo	Clinical efficacy, TDS (before and after treatment)	No follow-up
Zhu, 2014^29^	RCT	NR	1	96	PD	Desogestrel and ethinylestradiol tablets cyclic regimen (21 + 7) for 3 months	TCM	PRI, VAS, PPI, effectiveness (before and 1, 2, 3 months after treatment)	No follow-up
Chu, 2013^30^	RCT	2012.3–2013.3	1	164	PD	Desogestrel and ethinylestradiol tablets cyclic regimen (21 + 7) for 3 months	NSAIDs	TDS, endometrial thickness (before and after treatment）	3 months
Wang, 2013^18^	RCT	2011.5–2012.12	1	90	PD	Drospirenone ethinylestradiol tablets cyclic regimen (21 + 7) for 3 months	Placebo	VAS (before and 1 month after treatment), assessment; curative effect (3 months after treatment), venous blood PGE2, PGF2-α level (before and after treatment）	No follow-up
Liu, 2013^24^	RCT	2011.6–2013.6	1	120	NR	Desogestrel and ethinylestradiol tablets cyclic regimen (21 + 7) for 1 month	COC + TCM	Recovery time of menstruation, total time of treatment, effectiveness and recurrence rate	3 months
Liu, 2013^31^	RCT	2007.10–2012.10	1	315	PD	Desogestrel and ethinylestradiol tablets cyclic regimen (21 + 7) for 3 months	COC + TCM/TCM	Total efficiency, effective rate, cure rate	6 months
Gan, 2024^32^	RCT	2019.6–2020.6	8	164	PD	Desogestrel and ethinylestradiol tablets cyclic regimen (21 + 7) for 3 months	TCM	VAS, CMSS, and level of PGF2a, PGE2, PGF2a/PGE2, NO (before and 4, 8, 12 weeks after treatment)	No follow-up
Wang, 2019^39^	RCT	2012.12–2016.3	1	87	SD	Ethinylestradiol and cyproterone acetate tablets continuous regimen and cyclic regimen (21 + 7) for 6 months	GnRH agonist	Clinical efficacy, pain scores, sex hormone levels, serum levels of CA125, bone mineral density, recurrence rates (before and after treatment)	12 months
Sun, 2016^40^	RCT	2012.4–2014.4	1	80	SD	Ethinylestradiol and cyproterone acetate tablets continuous regimen for 6 months	COC + TCM	Dysmenorrheal rate, chronic pelvic pain rate, pain during sex rate, level of HDL/LDL, HDL/TG, HDL/TC, HDL-C, CA125 (before and after treatment)	12 months
Wang, 2021^41^	Cohort	2018.1–2021.1	1	62	SD	Drospirenone and ethinylestradiol tablets cyclic regimen (21 + 7) for 6 months	COC + TCM	VAS, pelvic mass diameter, CA125 level (before and after treatment)	No follow-up
Kong, 2017^19^	Non-RCT	2014.2–2016.2	1	316	SD	Drospirenone and ethinylestradiol tablets or ethinylestradiol and cyproterone acetate tablets cyclic regimen (NR) for 6 or 12 months	No treatment	Dysmenorrhea VAS score (before and every 6 months after treatment)	18 months
Yin, 2023^42^	RCT	2019.8–2022.9	1	150	SD	Drospirenone and ethinylestradiol tablets cyclic regimen (NR) for 6 months	COC + TCM	Clinical efficacies, symptom disappearance time, maximum diameter of the lesion, uterine volume, IL-8, VEGF, NLR (before and after treatment)	No follow-up
Kang, 2015^43^	RCT	2010.2–2014.1	1	74	SD	Drospirenone and ethinylestradiol tablets continuous regimen or cyclic regimen (21 + 7) for 6 or 12 months	/	Clinical symptoms, recurrence (after treatment)	>24 months^a^
Chen, 2017^44^	RCT	2015.3–2016.3	1	121	SD	Drospirenone ethinylestradiol tablets cyclic regimen (21 + 7) for 3 months	COC + TCM	Clinical efficacy, CA125 levels, dysmenorrhea scores, pelvic mass volume, pain, potential generandi, recurrence rates (before and after treatment)	No follow-up
Lu, 2019^45^	RCT	2017.2–2018.11	1	108	SD	Drospirenone ethinylestradiol tablets cyclic regimen (21 + 7) for 3 months	COC + TCM	Clinical efficacy, cytokine levels, pain, mass diameter (before and after treatment)	No follow-up
Liu, 2018^46^	RCT	2013.1–2017.10	1	82	SD	Drospirenone ethinylestradiol tablets continuous regimen for 6 months	COC + TCM	Clinical efficacy, VAS scores, pelvic mass diameter, uterine volume, E2 level, PGE2 level, hemorrheology indexes, EHP-5 scores (before and after treatment)	6 months
Li, 2016^47^	RCT	2014.7–2015.7	1	86	SD	Ethinylestradiol and cyproterone acetate tablets cyclic regimen (21 + 7) for 3 months	GnRH agonist	Curative effect, level of CA125, FSH, LH, E2 (before and after treatment)	6 months
Li, 2017^48^	Cohort	2007.1–2016.9	1	102	SD	Ethinylestradiol and cyproterone acetate tablets cyclic regimen (21 + 7) with follow-up visiting 12–84 months	ING-IUS	Variations in endometriosis-related pain, sexual function, VAS, FSFL, and SF-36 (before and 3, 6, 12, 24 months after treatment)	12–84 months^a^
Wang, 2018^49^	Non-RCT	2016.10–2017.9	1	84	SD	Ethinyl estradiol cyproterone tablets cyclic regimen (21 + 7) for 4 months	GnRH agonist	Clinical efficacy, incidence of dysmenorrhea, changes of serum estradiol, progesterone, luteinizing hormone, TGF-β, IL-4, IL-10, IL-17 levels (before and after treatment)	No follow-up

^a^ Studies enrolling patients with SD after surgery for endometriosis did not report the time of treatment cessation, instead, length from time of surgery to last visit was reported.

21 + 7, 21 days of COC with 7 days of no treatment; CA, cancer antigen; PGF-2α, prostaglandin F2 alpha; CMSS, Cox menstrual symptom scale; EHP-5, endometriosis health profile-5; FSFI, female sexual function index; GSH-Px, glutathione peroxidase; HDL, high-density lipoprotein cholesterol; IL-10, interleukin-10; LDL, low-density lipoprotein cholesterol; LNG-IUS, levonorgestrel-releasing intrauterine system; NLR, neutrophil–lymphocyte ratio; NO, nitric oxide; NSAIDs, nonsteroidal anti-inflammatory drugs; PD, primary dysmenorrhea; PPI, present pain intensity; PRI, pain rating index; RCT, randomized controlled study; SD, secondary dysmenorrhea; SF-36, short form 36-item health survey; SOD, superoxide dismutase; CAT, catalase CAT; TC, total cholesterol; TCM, traditional Chinese medicine; TDS, total dysmenorrhea score; TG, triglycerides; TGF-β, transforming growth factor beta; VAS, visual analogue scale; VEGF, vascular endothelial growth factor.

### Risk of bias

**FIG. 2.**
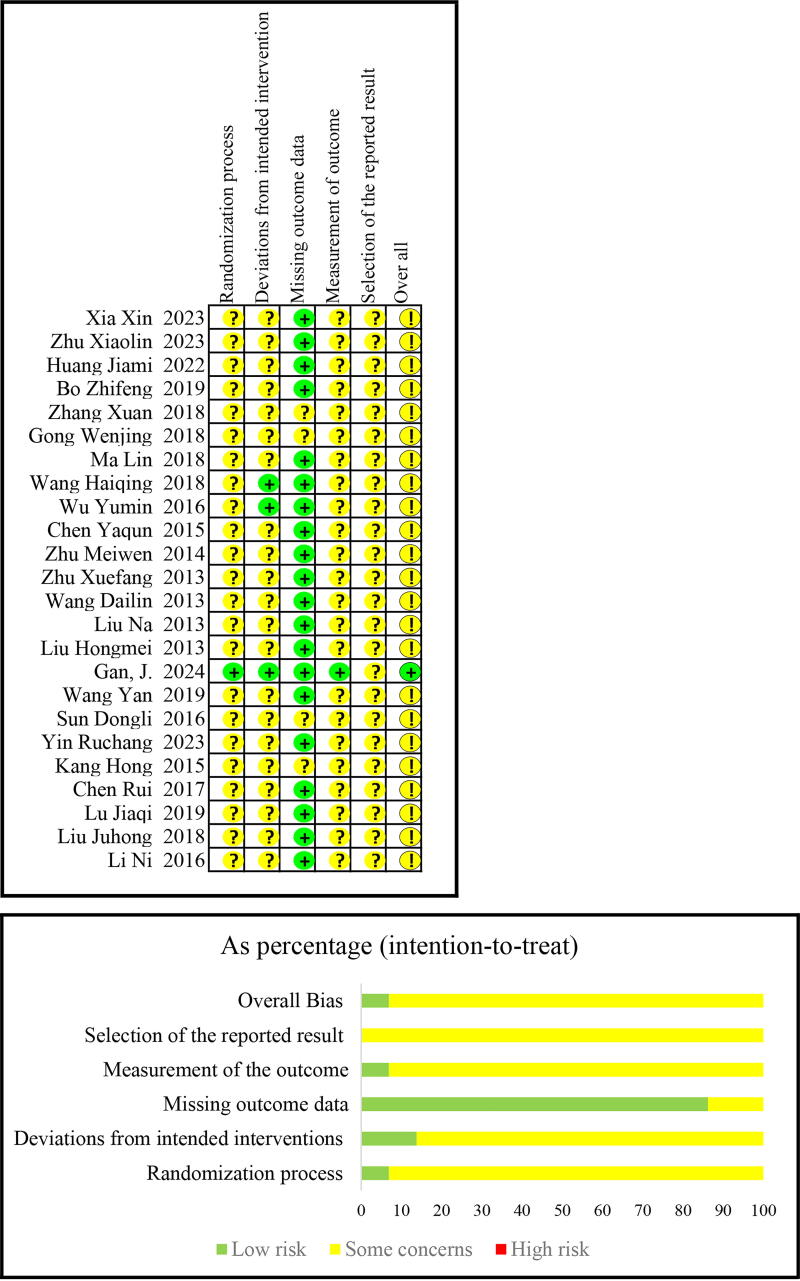
Risk of bias: Cochrane Risk of Bias tool for RCTs. Bias is assessed as a judgment (high, low, or unclear) for individual elements from five domains. RCT, randomized controlled trial.

Among the 24 RCTs, 1 trial^32^ had a low risk of bias, and the remaining 23^18,24–31,33–40,42–47^ had an unclear risk of bias because the methodology was not reported in depth. All RCTs used random allocation, but only one trial^32^ described the allocation concealment mechanism. This trial was also the only one that described double-blinding of personnel and study subjects (Fig. 2). The overall risk of bias for the two non-RCTs^19,49^ was moderate; their bias due to deviations from intended interventions and missing data was moderate, and their bias in measurement of outcomes was serious (Supplementary Tables S1 and S2). The two cohort studies^41,48^ were considered high quality (score = 7/9 on the NOS). In these studies, bias may have been present in the outcome measurements, as VAS pain score, a self-reported outcome, was used, and the number of subjects lost to follow-up was not reported (Supplementary Tables S1 and S2).

### Study designs

#### COC dosing schedule

COCs were mainly used in a cyclic (21/7 formulation) rather than a continuous fashion (92.9% [*n* = 26/28 studies]^18,24–39,41–44,46–50^ vs. 14.3% [*n* = 4/28 studies]^39,40,43,45^), even for SD (88.9% [*n* = 16/18 studies]^19,33–39,41–44,46–49^ vs. 22.2% [*n* = 4/18 studies]^39,40,43,45^). The duration of COC treatment varied widely, ranging from 1 to 12 months, and it was longer and more variable for SD than PD (4 [1–12] vs. 3 [3–3] months) (Table 2).

#### Comparator

Active controls such as TCM or TCM combined with COCs were common comparators in studies of PD (66.7% [*n* = 6/9 studies])^25–27,29,31,32^ and SD (61.1% [*n* = 11/18 studies]).^33–36,38,40–42,44–46^ Negative controls (placebo and no treatment) were rarely used (PD: 22.2% [*n* = 2/9 studies]^28,31^; SD: 5.6% [*n* = 1/18 studies]).^19^ In PD, the negative controls were used in RCTs.^28,31^ In SD, a negative control was assigned according to patient choice in a non-RCT^19^ (Table 2).

#### Type and severity of dysmenorrhea, outcome measures, and length of posttreatment follow-up

Most patients had moderate (64.7% [34.6%–76.5%]) or severe dysmenorrhea (33.1% [23.5%–35.3%]), and patients with mild dysmenorrhea were rarely enrolled.^19,25–27,31,32^ The majority of studies reported intensity of dysmenorrhea symptoms as an outcome (*n* = 22/28),^18,19,25,26,28–30,32–39,41,42,44–46,48,49^ most commonly measured with the VAS pain score (59.1% [*n* = 13/22 studies]),^18,19,25,32,35,37–39,41,44–46,48^ followed by the Cox menstrual symptom scale (CMSS, 18.2% [*n* = 4/22 studies]).^25,32,34,35^ Most studies (53.6% [*n* = 15/28 studies]^19,24–27,30,31,33,38–40,46,47^; PD: 55.6% [*n* = 5/9 studies]^25–27,30,31^; SD: 50.0% [*n* = 9/18 studies]^19,33,38–40,43,46–48^) had a posttreatment follow-up varying from 3 to 18 months. The median length of posttreatment follow-up was longer for SD than PD (6 [3–18] vs. 3 [3–6] months) (Table 2).

### COC effectiveness

#### Pre- versus post-COC treatment

Scores on all dysmenorrhea symptom measures were significantly decreased after 2–24 months of COC treatment in all studies (Table 3). Median reductions in VAS pain scores from pre- to post-COC treatment were 2.375 [1.31–3.2] and 2.81 [2–5.5] for PD and SD, respectively. Pooled estimates of VAS pain scores pre- and post-COC treatment are presented in Supplementary Data (Supplementary Figs. S1–S3).

**Table 3.** Effectiveness: pre- versus post-COC Treatment

Study	Baseline	Posttreatment						
		1 month	2 months	3 months	4 months	6 months	12 months	24 months
Primary dysmenorrhea								
VAS, 0–10 scale								
Gan, 2024^32^	5.08	2.53**	2.01**	1.88**	/	/	/	/
Zhu, 2014^29^	4.77	4.29	3.93*	3.11*	/	/	/	/
Bu, 2019^25^	6.28	/	/	3.19*	/	/	/	/
Wang, 2013^18^	4.63	3.32*	/	/	/	/	/	/
CMSS, 0–72 scale								
Gan, 2024^32^	30.34	14.82**	14.32**	13.85**	/	/	/	/
Bu, 2019^25^	22.89	/	/	10.30*	/	/	/	/
TDS, 0–6 scale								
Chen, 2015^28^	3.8	/	/	1.1, NR	/	/	/	/
Chu, 2013^30^	3.88	/	/	1.71*	/	/	/	/
SF-MPS (PRI, 0–33 scale)								
Zhu, 2014^29^	12.17	11.28	10.89*	9.53*	/	/	/	/
SF-MPS (PPI, 0–4 scale)								
Zhu, 2014^29^	2.14	1.99	1.71*	1.58*	/	/	/	/
CR-NCM, 0–15 scale								
Zhang, 2018^26^	12.06	/	/	7.02***	/	/	/	/
Secondary dysmenorrhea								
VAS, 0–10 scale								
Li, 2017^48^	7.6	/	/	2.7**		2.6**	2.3**	2.1**
Kong, 2017^19^	7	/	/	/	/	4*	5*	/
Wang, 2018^37^	8.02	/	/	/	/	3.86*	/	/
Wang, 2019^39^	5.45	/	/	/	/	2.97***	/	/
Wang, 2021^41^	4.56	/	/	/	/	2.54*	/	/
Liu, 2018^46^	6.83	/	/	/	/	2.07*	/	/
Wang, 2019^39^	5.43	/	/	/	/	0.84***	/	/
Wu, 2016^38^	7.433	8.200, NR	5.700, NR	5.367*	/	/	/	/
Huang, 2022^35^	6.17	/	/	3.36***	/	/	/	/
Chen, 2017^44^	7.19	/	/	4.87*	/	/	/	/
Lu, 2019^45^	7.07	/	/	4.10*	/	/	/	/
CMSS, 0–72 scale								
Zhu, 2023^34^	19.97	16.34**	12.66**	9.48**	/	/	/	/
Huang, 2022^35^	25.5	/	/	20.5**	/	/	/	/
TDS, 0–6 scale								
Wang, 2018^49^	4.69	/	/	/	3.26*	/	/	/
CR-NCM, 0–15 scale								
Wu, 2016^38^	10.100	8.200**	6.833**	6.233 **	/	/	/	/
Xia, 2023^33^	12.17	/	/	8.92 *	/	/	/	/
POGS, 0–16 scale								
Gong, 2018^36^	11.4	6.8*	/	/	/	/	/	/

Results are presented as mean values for all studies except Kong et al. (2017), for which median values are shown. One study did not report the statistical significance of within-group changes from baseline (Chen et al., 2015).

Significant difference versus baseline: *p < 0.05; **p < 0.01; ***p < 0.001.

CR-NCM, Guidelines for Clinical Research on New Chinese Medicines; NR, not reported; POGS, Practical Obstetrics and Gynecology Standards; SF-MPS, short-form McGill pain scale; VAS, visual analogue scale.

#### COCs versus comparators

For PD, COC treatment was more effective than placebo^18,28^ and comparable with NSAIDs^30^ for reducing symptoms of dysmenorrhea. Findings for COCs compared with TCM were inconsistent. One RCT^29^ reported that TCM was more effective than COCs for reducing symptoms of dysmenorrhea, while another RCT^32^ reported that COCs were significantly more effective than TCM.

For SD, two RCTs showed that COCs and TCM were comparable for reducing symptoms of dysmenorrhea,^36,38^ while another three RCTs showed that TCM was more effective than COCs.^33–35^ Two studies^39,49^ that compared COCs versus GnRH agonists showed that GnRH agonists were more effective than cyclic COCs for reducing symptoms of dysmenorrhea; however, another study^39^ reported that continuous COCs were comparable with GnRH agonist. For COCs versus LNG-IUS, one RCT reported that COCs were comparable with LNG-IUS for reducing symptoms of dysmenorrhea,^37^ while a retrospective cohort study reported that LNG-IUS was more effective than COCs^48^ (Table 4).

**Table 4.** Effectiveness: COCs Versus Comparators

Study	Scale	Intervention	Baseline	Posttreatment						
				1 month	2 months	3 months	4 months	6 months	12 months	24 months
Primary dysmenorrhea										
COC vs. placebo										
Wang, 2013^31^	VAS	COC cyclic	4.63	3.32*	/	/	/	/	/	/
		Placebo	4.78	4.15	/	/	/	/	/	/
Chen, 2015^28^	TDS	COC cyclic	3.8	/	/	1.1**	/	/	/	/
		Placebo	3.7	/	/	2.8	/	/	/	/
COC vs. NSAIDs										
Chu, 2013^30^	TDS	COC cyclic	3.88	/	/	1.71	/	/	/	/
		NSAIDs	3.91	/	/	1.86	/	/	/	/
COC vs. TCM										
Gan, 2024^32^	VAS	COC cyclic	5.08	2.53**	2.01***	1.88**	/	/	/	/
		TCM	5.35	3.59	2.87	2.54	/	/	/	/
	CMSS	COC cyclic	30.34	14.82***	14.32***	13.85***	/	/	/	/
		TCM	32.1	27.02	24.73	24.10	/	/	/	/
Zhu, 2014^29^	VAS	COC cyclic	4.77	4.29	3.93*	3.11*	/	/	/	/
		TCM	4.78	4.32	3.66	2.83	/	/	/	/
	SF-MPS (PRI)	COC cyclic	12.17	11.28	10.89*	9.53*	/	/	/	/
		TCM	12.21	11.51	10.02	8.45	/	/	/	/
	SF-MPS (PPI)	COC cyclic	2.14	1.99	1.71	1.58*	/	/	/	/
		TCM	2.27	2.08	1.55	1.23	/	/	/	/
Secondary dysmenorrhea										
COC vs. TCM										
Huang, 2022^35^	VAS	COC cyclic	6.17	/	/	3.36	/	/	/	/
		TCM	6.56	/	/	3.76	/	/	/	/
	CMSS	COC cyclic	25.5	/	/	20.5***	/	/	/	/
		TCM	23	/	/	17.0	/	/	/	/
Wu, 2016^38^	VAS	COC cyclic	7.433	8.200***	5.700***	5.367***	/	/	/	/
		TCM	7.333	3.767	2.267	1.200	/	/	/	/
	CR-NCM	COC cyclic	10.1	8.200	6.833*	6.233**	/	/	/	/
		TCM	10.8	7.033	5.133	3.667	/	/	/	/
Zhu, 2023^34^	CMSS	COC cyclic	19.97	16.34	12.66*	9.48**	/	/	/	/
		TCM	20.18	15.19	10.48	4.56	/	/	/	/
Xia, 2023^33^	CR-NCM	COC cyclic	12.17	/	/	8.92*	/	/	/	/
		TCM	11.85	/	/	6.4	/	/	/	/
Gong, 2018^36^	POGS	COC cyclic	11.4	6.8, NR	/	/	/	/	/	/
		TCM	11.7	6.1	/	/	/	/	/	/
COC vs. GnRH agonist										
Wang, 2019^39^	VAS	COC cyclic	5.45	/	/	/	/	2.97*	/	/
		COC continuous	5.43	/	/	/	/	0.84	/	/
		GnRH agonist	5.39	/	/	/	/	0.60	/	/
Wang, 2018^49^	TDS	COC cyclic	4.69	/	/	/	3.26*	/	/	/
		GnRH agonist	4.72	/	/	/	1.63	/	/	/
COC vs. LNG-IUS										
Wang, 2018^37^	VAS	COC cyclic	8.02	/	/	/	/	3.86, NR	/	/
		LNG-IUS	7.64	/	/	/	/	3.72	/	/
Li, 2017^48^	VAS	COC cyclic	7.7		/	2.7	/	2.6*	2.3*	2.1*
		LNG-IUS	7.6		/	2.5	/	2.0	1.9	1.6

Significant difference versus comparators: *p < 0.05; **p < 0.01; ***p < 0.001.

### Safety

The AE incidence rates associated with the use of COCs were reported in 13 studies.^25,26,28,37,39,40,42–47,49^ The top five most frequently reported AEs were abnormal vaginal bleeding (*n* = 9 studies),^26,28,37,43–47,49^ breast pain (*n* = 8 studies),^25,26,28,37,40,42–44^ nausea or vomiting (*n* = 8 studies),^25,26,28,39,40,42,45,46^ headache (*n* = 7 studies),^25,26,40,42–45^ and change of mood (*n* = 3 studies) (Table 5). No deaths or thromboembolic events were reported after the use of COCs. Abnormal vaginal bleeding was the most frequently reported AE associated with the use of COCs, with incidence rates ranging from 2.4% to 51.4%,^26,28,43–47,49^ and significantly higher incidence rates in patients who used COCs in a continuous fashion compared with a cyclic fashion (51.4% vs. 19.4%).^43^ COC discontinuation ranged from 0.0% to 30.2%^27,29,32,37,38,43^ and was higher in patients with PD (30.1%,^27^ 17.1%,^29^ and 30.2%^32^) compared with SD (0.0%–5.3%^19,37,38,43^). In one study, patient drop-out factors included fear of unsafe hormone use (*n* = 28/113 patients, 24.8%), abnormal vaginal bleeding (*n* = 3/113 patients, 2.7%), fertility plan (*n* = 2/113 patients, 1.8%), and uncomfortable symptoms (*n* = 1/113 patient, 0.9%).^27^

**Table 5.** Safety Profiles of COCs

Adverse reaction	Study	COC dosing scheme	Incidence rate			
			3 months	4 months	6 months	12 months
Menstrual changes						
Abnormal vaginal bleeding	Chen, 2017^44^	Cyclic (21 + 7)	3/60, 5.0%			
	Lu, 2019^45^	Continuous	4/54, 7.4%			
	Liu, 2018^46^	Cyclic (NR)			2/41, 4.9%	
	Kang, 2015^43^	Continuous				18/35, 51.4%
		Cyclic (21 + 7)				7/36, 19.4%
	Li, 2016^47^	Cyclic (21 + 7)	16/43, 37.2%			
	Wang, 2018^49^	Cyclic (21 + 7)		1/42, 2.4%		
	Zhang, 2018^26^	Cyclic (21 + 7)	2/51, 3.9%			
	Chen, 2015^28^	Cyclic (21 + 7)	3/49, 6.1%			
	Wang, 2018^37^	Cyclic (21 + 7)			8/48, 16.7%	
Decrease in menstrual flow	Chen, 2015^28^	Cyclic (21 + 7)	9/49, 18.4%			
Menopause	Chen, 2017^44^	Cyclic (21 + 7)	2/60, 3.3%			
Breast pain	Bu, 2019^25^	Cyclic (21 + 7)	1/64, 1.6%			
	Zhang, 2018^26^	Cyclic (21 + 7)	2/51, 3.9%			
	Chen, 2015^28^	Cyclic (21 + 7)	5/49, 10.2%			
	Chen, 2017^44^	Cyclic (21 + 7)	2/60, 3.3%			
	Wang, 2018^37^	Cyclic (21 + 7)			1/48, 2.1%	
	Sun, 2016^40^	Continuous			16/40, 40.0%	
	Yin, 2023^42^	Cyclic (NR)			1/75, 1.3%	
	Kang, 2015^43^	Cyclic (21 + 7)				3/36, 8.3%
		Continuous				2/35, 5.7%
Nausea or vomiting	Bu, 2019^25^	Cyclic (21 + 7)	1/64, 1.6%			
	Zhang, 2018^26^	Cyclic (21 + 7)	4/51, 7.8%			
	Chen, 2015^28^	Cyclic (21 + 7)	3/49, 6.1%			
	Lu, 2019^45^	Continuous	3/54, 5.6%			
	Wang, 2019^39^	Continuous			3/30, 10.0%	
		Cyclic (21 + 7)			2/28, 7.1%	
	Sun, 2016^40^	Continuous			16/40, 40.0%	
	Yin, 2023^42^	Cyclic (NA)			1/75, 1.3%	
	Liu, 2018^46^	Cyclic (NA)			2/41, 4.9%	
Headache	Bu, 2019^25^	Cyclic (21 + 7)	1/64, 1.6%			
	Zhang, 2018^26^	Cyclic (21 + 7)	1/51, 2.0%			
	Chen, 2017^44^	Cyclic (21 + 7)	3/60, 5.0%			
	Lu, 2019^45^	Continuous	1/54, 1.9%			
	Sun, 2016^40^	Continuous			4/40, 10.0%	
	Yin, 2023^42^	Cyclic (NR)			1/75, 1.3%	
	Kang, 2015^43^	Cyclic (21 + 7)				2/36, 5.6%
		Continuous				1/35, 2.9%
Change of mood	Lu, 2019^45^	Continuous	4/54, 7.4%			
	Sun, 2016^40^	Continuous			14/40, 35.0%	
	Liu, 2018^46^	Cyclic (NR)			3/41, 7.3%	
Pruritus	Wang, 2019^39^	Continuous			2/30, 6.7%	
		Cyclic (21 + 7)			1/28, 3.6%	
	Yin, 2023^42^	Cyclic (NR)			1/75, 1.3%	
Weight gain	Chen, 2017^44^	Cyclic (21 + 7)	3/60, 5.0%			
	Wang, 2018^37^	Cyclic (21 + 7)			1/48, 2.1%	
Diarrhea	Yin, 2023^42^	Cyclic (NR)			1/75, 1.3%	
Hot flashes	Wang, 2018^49^	Cyclic (21 + 7)		2/42, 4.8%		
Acne	Wang, 2018^37^	Cyclic (21 + 7)			1/48, 2.1%	
Hyperhidrosis	Wang, 2018^49^	Cyclic (21 + 7)		3/42, 7.1%		
Change of sexual desire	Sun, 2016^40^	Continuous			14/40, 35.0%	
Others^a^	Kang, 2015^43^	Continuous				2/35, 5.7%
		Cyclic (21 + 7)				1/36, 2.8%

^a^ Others included weight gain and acne.

21 + 7, administrating 21 days of COC with 7 days of no treatment.

## Discussion

### Main findings

This systematic review summarized the designs of clinical studies and evaluated the effectiveness and safety of COCs for dysmenorrhea in Chinese women. Findings showed that most clinical studies investigating COCs in this population gave COCs in a cyclic fashion (21/7 formulation), used active controls, had 3 or 6 months of posttreatment follow-up, and measured the intensity of dysmenorrhea symptoms using the VAS pain score. COCs appeared to significantly reduce symptoms of dysmenorrhea in PD and SD, but insufficient data exist to compare the effectiveness of COCs to NSAIDs, TCM, GnRH agonists, and LNG-IUS. Abnormal menstrual bleeding was the most common AE associated with use of COCs, with an incidence that was significantly higher in patients who used COCs in a continuous fashion compared with a cyclic fashion.

### Strengths and limitations

This review had a robust sample size on which to base conclusions and included studies that provided an accurate representation of Chinese women with dysmenorrhea. The systematic search identified 28 relevant studies (24 RCTs, 2 non-RCTs, and 2 retrospective cohort studies) that included 3409 Chinese patients with PD or SD. The methodological quality of the included studies was considered acceptable. Most of the studies were RCTs, which are among the highest levels of scientific evidence, the non-RCTs were assessed as moderate risk of bias, and the retrospective cohort studies were considered high quality.

There were some potential biases in the review process. First, studies with <30 patients treated with COCs were excluded, potentially leading to selection bias. Second, most RCTs included in the review were assessed as unclear risk of bias using the Cochrane Risk of Bias tool. These RCTs may have had critical errors in design, analysis, or reporting that invalidated their results. Third, treatment effect in the included studies was mainly assessed with VAS pain scores; however, decreases in pain scores from baseline can be misleading because of the placebo effect, which often produces a high response rate. Last, evidence synthesis used a narrative rather than a quantitative approach, which precluded making statistical claims from numerical data.

### Interpretation

To the authors’ knowledge, this is the first review to summarize the designs of clinical studies and describe the effectiveness and safety of COCs for PD and SD in the Chinese population. With regard to study design, the clinical studies included in this review investigated PD or SD, but no single study included both, likely because management of PD and SD differs. Placebo or no-treatment controls were rarely used, especially for patients with SD, likely because they suffered from moderate to severe dysmenorrhea (median VAS score 7.0 [4.5–8.0]^19,35,37–39,41,44–46,48^ at baseline), their treatment needs were high, and creating placebo/no-treatment groups would have been unethical. Active controls were used, but selection may have been challenging, as a gold standard treatment for dysmenorrhea has not been identified in China. Chinese investigators preferred TCM, which is a common choice among patients with dysmenorrhea in Chinese clinical practice.^51^ All clinical studies in PD gave COCs in a cyclic fashion (21/7 formulation) for 3 months. These results imply that Chinese practitioners prefer this dosing scheme. The duration of treatment in SD was longer than in PD, likely because extended COC treatment may benefit the primary gynecologic diseases such as endometriosis and adenomyosis that underlie SD.

With regard to the effectiveness of COCs for dysmenorrhea in the Chinese population, two RCTs showed that COCs were more effective than placebo for reducing pain in dysmenorrhea.^28,31^ These results are consistent with those from a previous review focused on SD.^52^ The methodologies of the clinical studies included in this review and the studies included in the previous review varied widely. The consistent findings across this methodologically diverse evidence strongly support the effectiveness of COCs for treating dysmenorrhea in Chinese women.

Data in the included studies were insufficient to reach conclusions about the effectiveness of COCs compared with other approaches. Only one RCT^30^ compared COCs with NSAIDs in PD, with more patients achieving a pain score of 0 after COC treatment, implying COCs may be more effective than NSAIDs. One well-designed double-blind RCT with a low risk of bias revealed that COCs were more effective than TCM for reducing pain scores in PD. Notably, all studies that compared COCs with TCM had a duration ≤3 months; therefore, the long-term effect of COCs versus TCM on dysmenorrhea has not been evaluated. GnRH agonists were more effective than cyclic COCs in reducing symptoms of dysmenorrhea^39,49^; this may be because GnRH agonists induce amenorrhea, which results in the complete resolution of dysmenorrhea. Continuous COCs may also induce amenorrhea and improve dysmenorrhea to a similar degree as GnRH agonists.^39^ One RCT reported that continuous use of COCs after laparoscopic surgery was more helpful in reducing the recurrence of dysmenorrhea and non-menstrual pain and prolonging the recurrence interval than cyclic COCs. However, continuous administration of COCs is associated with a high incidence of abnormal vaginal bleeding,^43^ which may cause patients to discontinue treatment. For COCs versus LNG-IUS, one RCT^37^ reported that COCs were comparable with LNG-IUS for dysmenorrhea, while a retrospective cohort study^48^ reported that LNG-IUS was more effective than COCs.

## Conclusion

This systematic review revealed that COC treatment is effective for PD and SD in the Chinese population with an acceptable safety profile. Continuous COCs may be more effective than cyclic COCs for SD; however, the use of continuous COCs increases the incidence of abnormal vaginal bleeding. Insufficient data exist to reach conclusions about the effectiveness of COC therapy compared with TCM, GnRH agonists, and LNG-IUS in the Chinese population. Additional well-designed, head-to-head, comparative trials are needed to clarify the role of COCs and other treatments.

## Data Availability

All data generated or analyzed during this study are included in this published article and its Supplementary Data.

## Abbreviations Used

COCs: combined oral contraceptives
GnRH: gonadotropin-releasing hormone
LNG-IUS: levonorgestrel-releasing intrauterine system
non-RCT: non-randomized controlled trial
NOS: Newcastle–Ottawa scale
NSAIDs: nonsteroidal anti-inflammatory drugs
PD: primary dysmenorrhea
PG: prostaglandin
PRISMA: Preferred Reporting Items for Systematic Reviews and Meta-Analyses
RCT: randomized controlled trial
SD: secondary dysmenorrhea
TCM: traditional Chinese medicines
VAS: visual analogue scale
