# Supplementary material for: Controlled Clinical Studies of Combined Oral Contraceptives for Dysmenorrhea in China: A Systematic Literature Review
Source: Womens Health Rep (New Rochelle). 2025 Sep 22;6(1):964–77. doi: 10.1177/26884844251379378 (PMC12547401; doi:10.1177/26884844251379378)
Supplement: Supplementary Appendix [file 26884844251379378_supplementary_appendix.docx]

| #1 | Dysmenorrhea[Title/Abstract] OR "Dysmenorrhea"[Mesh] | 7,976 |
| --- | --- | --- |
| #2 | ("Contraceptive Agents"[Mesh] OR Contraceptive[Title/Abstract]) OR ("ethinyl estradiol-desogestrel combination" [Supplementary Concept] OR Mercilon[Title/Abstract] OR "EE-DSG"[Title/Abstract]) OR ("Desogestrel"[Mesh] OR Marvelon[Title/Abstract] OR Mawelon[Title/Abstract] OR Desogestrel[Title/Abstract]) OR ("Ethinyl Estradiol"[Mesh] OR (Deoxyprogesterone[Title/Abstract] AND ethinylestradio*[Title/Abstract])) OR ("Cyproterone acetate, ethinyl estradiol drug combination" [Supplementary Concept] OR Diane[Title/Abstract]) OR (Ethinylestradiol [Title/Abstract] AND Cyproterone[Title/Abstract]) OR ("drospirenone and ethinyl estradiol combination" [Supplementary Concept] OR Yasmin[Title/Abstract]) OR (Drospirenone[Title/Abstract] AND Ethinylestradiol[Title/Abstract]) | 81,832 |
| #3 | China[Affiliation] OR Chinese[Affiliation] | 2,850,393 |
| #4 | "2003/01/01"[Date - Publication]: "2024/01/19"[Date - Publication] | 8,804,497 |
| #5 | #1 AND #2 AND #3 AND #4 | 8 |
| #6 | #5 Filters: Clinical Study, Clinical Trial, Clinical Trial, Phase III, Clinical Trial, Phase IV, Comparative Study, Meta-Analysis, Observational Study, Pragmatic Clinical Trial, Randomized Controlled Trial, Review, Systematic Review | 7 |

**Appendix S1** Search strategy in PubMed

**Appendix S2** Search strategy in Embase

| #1 | 'dysmenorrhea':ta,ab,kw OR 'dysmenorrhea'/exp | 17,873 |
| --- | --- | --- |
| #2 | ('contraceptive agent'/exp OR 'contraceptive':ta,ab,kw) OR ('desogestrel plus ethinylestradiol'/exp OR Mercilon:ta,ab,kw OR 'EE-DSG':ta,ab,kw) OR ('desogestrel'/exp OR Marvelon:ta,ab,kw OR Mawelon:ta,ab,kw OR 'desogestrel':ta,ab,kw) OR ('ethinylestradiol'/exp OR Deoxyprogesterone:ta,ab,kw AND ethinylestradio*:ta,ab,kw) OR ('cyproterone acetate plus ethinylestradiol'/exp OR Diane:ta,ab,kw) OR (Ethinylestradiol:ta,ab,kw AND Cyproterone:ta,ab,kw) OR ('drospirenone plus ethinylestradiol'/exp OR Yasmin:ta,ab,kw) OR (Drospirenone:ta,ab,kw AND Ethinylestradiol:ta,ab,kw) | 197,229 |
| #3 | 'China':ff OR 'Chinese':ff | 1,310,536 |
| #4 | [2003-2024]/py | 27,734,093 |
| #5 | #1 AND #2 AND #3 AND #4 | 62 |
| #6 | #5 ('case control study'/de OR 'clinical article'/de OR 'clinical study'/de OR 'clinical trial'/de OR 'clinical trial topic'/de OR 'cohort analysis'/de OR 'comparative effectiveness'/de OR 'comparative study'/de OR 'controlled clinical trial topic'/de OR 'controlled study'/de OR 'correlational study'/de OR 'cross sectional study'/de OR 'data collection method'/de OR 'double blind procedure'/de OR 'drug dose comparison'/de OR 'evidence based medicine'/de OR 'feasibility study'/de OR 'human'/de OR 'intervention study'/de OR 'longitudinal study'/de OR 'major clinical study'/de OR 'medical record review'/de OR 'meta analysis'/de OR 'methodology'/de OR 'multicenter study'/de OR 'multicenter study topic'/de OR 'observational study'/de OR 'pragmatic trial'/de OR 'proportional hazards model'/de OR 'prospective study'/de OR 'quality control'/de OR 'questionnaire'/de OR 'randomized controlled trial'/de OR 'randomized controlled trial topic'/de OR 'retrospective study'/de OR 'study design'/de OR 'systematic review'/de OR 'telephone interview'/de OR 'validation study'/de) AND [female]/lim AND ('article'/it OR 'article in press'/it OR 'review'/it) | 48 |

**Appendix S3** Search strategy in CNKI

| #1 | （篇关摘：痛经 + 痛经发生率 + 原发性痛经 + 子宫内膜异位症痛经 +改善痛经 + 重度痛经(精确)） | 9,038 |
| --- | --- | --- |
| #2 | （篇关摘：口服避孕药 + 口服避孕药物 + '口服避孕药(coc)'(精确)）OR（篇关摘：妈富隆 + 妈富隆片 + '去氧孕烯炔雌醇片(妈富隆)' + '避孕药(妈富隆)' + 避孕药妈富隆治疗(精确)）OR（篇关摘：美欣乐 + 欣妈富隆(精确)）OR（篇关摘：去氧孕烯炔雌醇 + 去氧孕烯炔雌醇片(精确)）OR（篇关摘：达英 + '达英-35' + 达英35(精确)）OR（篇关摘：环丙孕酮 + 环丙孕酮片 + 环丙孕酮醋酸酯 +炔雌醇环丙孕酮(精确)）OR（篇关摘：优思明 + 优思明片 + '优思明(屈螺酮炔雌醇)' + 优思明短效口服避孕药 + 屈螺酮炔雌醇片（II）(精确)）OR（篇关摘：屈螺酮炔雌醇 + 屈螺酮炔雌醇片 + 屈螺酮炔雌醇复合片 + 屈螺酮炔雌醇避孕药 + '屈螺酮炔雌醇(ii)'(精确)） | 9,975 |
| #3 | #1 AND #2 | 133 |
| #4 | #3 from 2003 to 2024 | 130 |

**Appendix S4** Search strategy in Wangfang

| #1 | (题名或关键词:(痛经) or 主题:(痛经) or 题名或关键词:(原发性痛经) 主题:(原发性痛经)) and 发表时间:2003-* | 29,361 |
| --- | --- | --- |
| #2 | (题名或关键词:(口服避孕药) or 主题:(口服避孕药) or 题名或关键词:(避孕药) or 主题:(避孕药) or 题名或关键词:(妈富隆) or 主题:(妈富隆) or 题名或关键词:(去氧孕烯炔雌醇) or 主题:(去氧孕烯炔雌醇) or 题名或关键词:(美欣乐) or 主题:(美欣乐) or 题名或关键词:(欣妈富隆) or 主题:(欣妈富隆) or 题名或关键词:(达英) or 主题:(达英) or 题名或关键词:(达英-35) or 主题:(达英-35) or 题名或关键词:(炔雌醇环丙孕酮) or 主题:(炔雌醇环丙孕酮) or 题名或关键词:(环丙孕酮) or 主题:(环丙孕酮) or 题名或关键词:(优思明) or 主题:(优思明) or 题名或关键词:(屈螺酮炔雌醇片（II）) or 主题:(屈螺酮炔雌醇片（II）) or 题名或关键词:(屈螺酮炔雌醇) or 主题:(屈螺酮炔雌醇)) and 发表时间:2003-* | 54,863 |
| #3 | #1 AND #2 from 2003 to 2024 | 648 |
| #4 | #3 AND 期刊论文 | 536 |

**Appendix S5** Search strategy in VIP

| #1 | M=痛经 OR M=原发性痛经and 发表时间:2003-* | 9,867 |
| --- | --- | --- |
| #2 | M=口服避孕药 OR M=避孕药 OR M=妈富隆 OR M=去氧孕烯炔雌醇 OR M=美欣乐 OR M=欣妈富隆 OR M=达英 OR M=达英-35 OR M=炔雌醇环丙孕酮 OR M=环丙孕酮 OR M=优思明 OR M=屈螺酮炔雌醇片（II） OR M=屈螺酮炔雌醇 and 发表时间:2003-* | 9,265 |
| #3 | #1 AND #2 from 2003 to 2024 | 108 |
| #4 | #3 AND 期刊论文 | 536 |

**Appendix S6** Search strategy in Chinese Clinical Trial Registry

| #1 | 正式科学名：痛经 | 69 |
| --- | --- | --- |

**Appendix S7** Search strategy in ClinicalTrials.gov

| #1 | Condition/disease: dysmenorrhea  Location: China | 15 |
| --- | --- | --- |
