# Supplementary material for: Controlled Clinical Studies of Combined Oral Contraceptives for Dysmenorrhea in China: A Systematic Literature Review
Source: Womens Health Rep (New Rochelle). 2025 Sep 22;6(1):964–77. doi: 10.1177/26884844251379378 (PMC12547401; doi:10.1177/26884844251379378)
Supplement: Supplementary Figures [file 26884844251379378_supplementary_figures.docx]

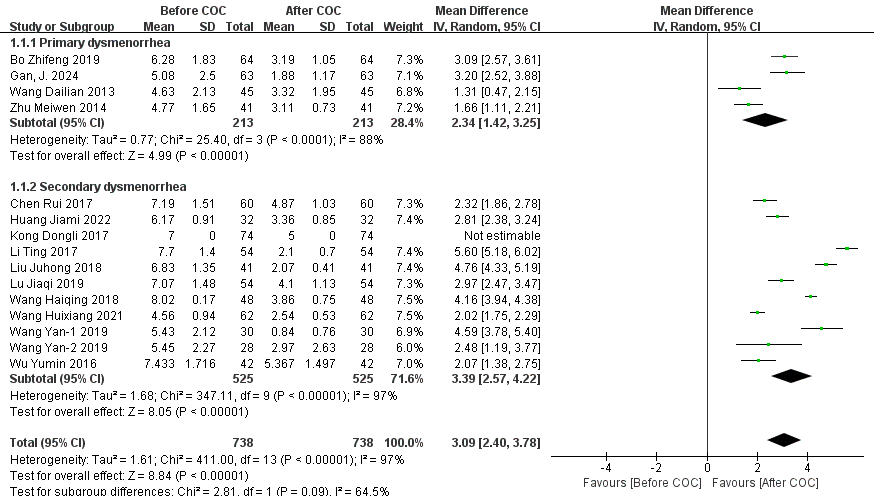


**Figure S1.** VAS pain scores pre- versus post-treatment with COCs

Note: Individual and pooled statistics were expressed as mean differences (MD) and 95% confidence intervals (CI). Cochrane Review Manager 5.4 was used to summarize effect estimates and generate forest plots. Statistical heterogeneity was assessed using Cochran’s Q test. If there was no substantial statistical heterogeneity (I^2^ < 50%), data were combined using a fixed-effect model; otherwise, a random-effect model was used. The cut-off for statistical significance was set at a two-sided p < 0.05. Results are presented as mean values for all studies except Kong Dongli et al., 2017, for which median values are shown. COC: combined oral contraceptive.


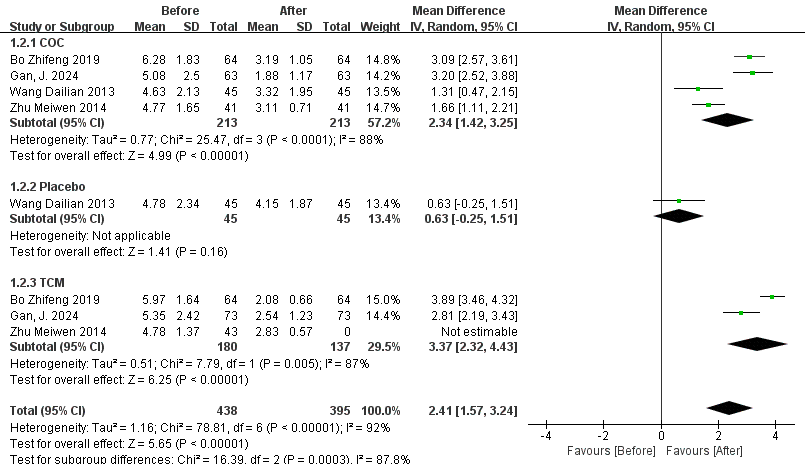


**Figure S2.** VAS pain scores pre- versus post-treatment with COCs, placebo, and TCM in patients with primary dysmenorrhea

Note: Individual and pooled statistics were expressed as mean differences (MD) and 95% confidence intervals (CI). Cochrane Review Manager 5.4 was used to summarize effect estimates and generate forest plots. Statistical heterogeneity was assessed using Cochran’s Q test. If there was no substantial statistical heterogeneity (I^2^ < 50%), data were combined using a fixed-effect model; otherwise, a random-effect model was used. The cut-off for statistical significance was set at a two-sided p < 0.05. COC: combined oral contraceptive; TCM: traditional Chinese medicine.

**
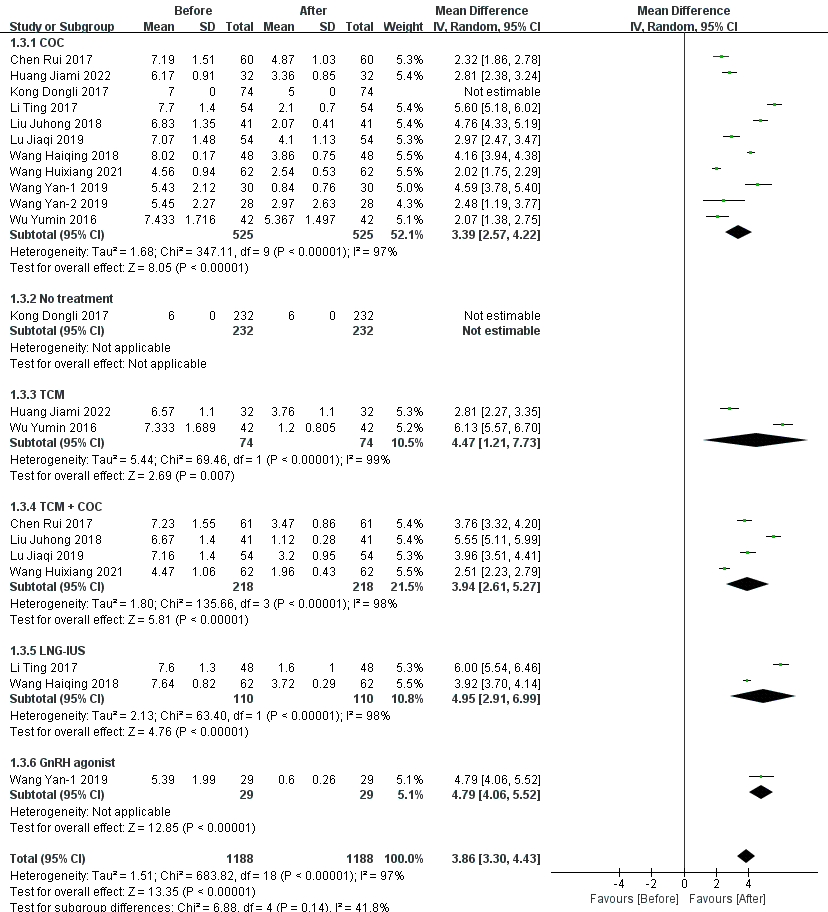
**

**Figure S3.** VAS pain score scores pre- versus post-treatment with COCs, no treatment, TCM, TCM + COC, LNG-IUS, and GnRH agonist in patients with secondary dysmenorrhea

Note: Individual and pooled statistics were expressed as mean differences (MD) and 95% confidence intervals (CI). Cochrane Review Manager 5.4 was used to summarize effect estimates and generate forest plots. Statistical heterogeneity was assessed using Cochran’s Q test. If there was no substantial statistical heterogeneity (I^2^ < 50%), data were combined using a fixed-effect model; otherwise, a random-effect model was used. The cut-off for statistical significance was set at a two-sided p < 0.05. Results are presented as mean values for all studies except Kong Dongli et al., 2017, for which median values are shown. COC: combined oral contraceptive; TCM: traditional Chinese medicine; LNG-IUS: Levonorgestrel intrauterine system; GnRH agonist: gonadotropin-releasing hormone agonist.
