# Supplementary material for: Controlled Clinical Studies of Combined Oral Contraceptives for Dysmenorrhea in China: A Systematic Literature Review
Source: Womens Health Rep (New Rochelle). 2025 Sep 22;6(1):964–77. doi: 10.1177/26884844251379378 (PMC12547401; doi:10.1177/26884844251379378)
Supplement: Supplementary Tables [file 26884844251379378_supplementary_tables.docx]

| Author, year | Bias due to confounding | Bias in selection of participants into the study | Bias in classification of interventions | Bias due to deviations from intended interventions | Bias due to missing data | Bias in measurement of outcomes | Bias in selection of the reported result | Overall bias |
| --- | --- | --- | --- | --- | --- | --- | --- | --- |
| Kong Dongli, 2017^[19]^ | Critical | Low | Low | Moderate | Moderate | Serious | Low | Moderate |
| Wang Lei, 2018^[49]^ | Low | Low | Low | Moderate | Moderate | Serious | Low | Moderate |

**Table S1.** Risk of Bias in Non-randomized Studies of Interventions (ROBINS-I) tool for non-RCTs

Based on answers to the signaling questions, judgements for each bias domain, and for overall risk of bias, can be ‘Low’, ‘Moderate’, ‘Serious’ or ‘Critical’ risk of bias.

**Table S2** Newcastle–Ottawa quality assessment scale (NOS) for cohort studies

| Author, year | Selection | | | | Comparability | Exposure | | | Final score |
| --- | --- | --- | --- | --- | --- | --- | --- | --- | --- |
|  | Is the case definition adequate | Representativeness of the cohort | Selection of Controls | Definition of Controls | Comparability of cases and controls on the basis of the design or analysis^*^ | Ascertainment of exposure | Same method of ascertainment for cases and controls | Non-Response rate |  |
| Wang Huixiang, 2021^[41]^ | ★ | ★ | ★ | ★ | ★★ | - | ★ | - | 7 |
| Li Ting, 2017^[48]^ | ★ | ★ | ★ | ★ | ★★ | - | ★ | - | 7 |

The Newcastle-Ottawa Scale provides a rating system ranging from 0 to 9 stars, with scores equal to or greater than 7 considered high quality and scores less than 7 considered low quality.

* A maximum of 2 stars could be awarded for this item. A study controlling for a dysmenorrhea symptom score received one star, and a study controlling for other major risk factors received an additional star.
